# Engineered adenovirus fiber shaft fusion homotrimer of soluble TRAIL with enhanced stability and antitumor activity

**DOI:** 10.1038/cddis.2016.177

**Published:** 2016-06-23

**Authors:** J Yan, L Wang, Z Wang, Z Wang, B Wang, R Zhu, J Bi, J Wu, H Zhang, H Wu, B Yu, W Kong, X Yu

**Affiliations:** 1 National Engineering Laboratory for AIDS Vaccine, School of Life Sciences, Jilin University, Changchun, Jilin, China; 2 Key Laboratory for Molecular Enzymology and Engineering of the Ministry of Education, School of Life Sciences, Jilin University, Changchun, Jilin, China

## Abstract

Successful cancer therapies aim to induce selective apoptosis in neoplastic cells. Tumor necrosis factor-related apoptosis-inducing ligand (TRAIL) is considered an attractive anticancer agent due to its tumor cell-specific cytotoxicity. However, earlier studies with recombinant TRAIL revealed many shortcomings, including a short half-life, off-target toxicity and existence of TRAIL-resistant tumor cells. In this study, we developed a novel engineering strategy for recombinant soluble TRAIL by redesigning its structure with the adenovirus knobless fiber motif to form a stable homotrimer with improved antitumor activity. The result is a highly stable fiber-TRAIL fusion protein that could form homotrimers similar to natural TRAIL. The recombinant fusion TRAIL developed here displayed high specific activity in both cell-based assays *in vitro* and animal tests *in vivo*. This construct will serve as a foundation for a new generation of recombinant proteins suitable for use in preclinical and clinical studies and for effective combination therapies to overcome tumor resistance to TRAIL.

Cancer therapies aim to induce selective apoptosis in neoplastic cells with limited concomitant side effects. Tumor necrosis factor (TNF)-related apoptosis-inducing ligand (TRAIL) is a promising agent for cancer therapy, as TRAIL-based therapeutics have been shown to be more effective inducers of apoptosis in cancer cells than in normal cells.^1, 2, 3^ Natural TRAIL is a type II membrane protein, and its membrane form is cleaved to produce a soluble, biologically active cytokine.^4^ The soluble TRAIL (sTR) protein exists as a homotrimer, and a single zinc atom is chelated by the Cys230 of each monomer. The trimeric structure is a critical requirement for its biological function.^5^ TRAIL induces apoptosis by binding to TRAIL-R1/death receptor 4 (DR4) and TRAIL-R2/death receptor 5 (DR5), which are widely expressed in cancer cells.^6, 7^ These interactions lead to trimerization of DR4/DR5 and their intracellular death domains and consequent recruitment of an adaptor molecule, Fas-associated death domain, which then signals tumor cell death via caspase-dependent apoptotic pathways.^8, 9^ Importantly, TRAIL induces apoptosis in malignant cells via both extrinsic and intrinsic pathways, and its effect is independent of the functional status of p53,^10, 11, 12, 13^ thus increasing the probability of the apoptotic outcome.^14^ However, TRAIL is unstable and has low activity, which are obvious shortcomings, and these deficiencies are further aggravated by a short half-life in the blood as well as the original and acquired resistance of certain cancers to TRAIL.^15^

The human adenovirus type 5 (Ad5) fiber protein is encoded by a single gene that expresses a polypeptide of 581 amino acid (aa) residues and exists as a homotrimer.^16^ The monomeric fiber protein is composed of an N-terminal tail of approximately 47 aa residues that interact with the penton base protein of the capsid, a shaft comprising 21 pseudo repeats of 15 aa and an ~180 aa C-terminal knob for cellular attachment. The formation of the trimeric fiber is less essential for its function and its assembly into virus particles. The fiber shaft at the C terminus was found to be required for fiber knob trimer formation, since knob proteins containing two or more shaft repeats can form functional trimers.^17^ Notably, formation of trimerization motifs by peptide fusion at the fiber C terminus has been reported.^18, 19, 20^ Another study demonstrated that a series of shortened fibers consisting of 6.5–9 shaft repeats, along with the tail, could be fused to the N terminus of the trimerization element.^21^

Here, to obtain a highly stable and active trimeric TRAIL protein while avoiding hepatocyte transduction *in vivo* by the fiber shaft,^22, 23^ several trimerization elements from human Ad5 and fowl Ad1 fiber were first used to engineer the sTR protein. A human Ad5 fiber sTR was reengineered to contain the N-terminal tail and the shortened shaft repeats (HA5FT) or to contain the C-terminal shaft repeat (HA5ST). A fowl Ad1 sTR (FA1FT) containing the full-length knobless fiber was also constructed. The results demonstrated that the reengineered HA5ST and FA1FT were stable trimers, displaying improved bioavailability and antitumor activity, whereas HA5FT was a nonfunctional fusion protein.

## Results

### Design, purification and characterization of trimeric adenovirus fiber-TRAIL fusion proteins

**Figure 1:**
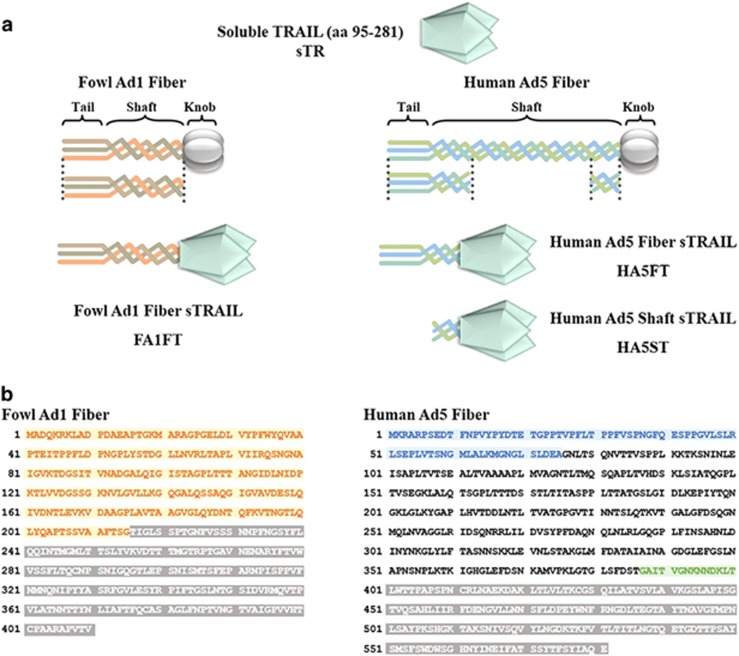
Design and amino acid sequence of recombinant TRAIL. (**a**) Schematic representation of TRAIL forms used in this study. (**b**) Amino acid sequence of knobless fiber from fowl Ad1 used in this study is colored in yellow, and the knob is colored in gray. Amino acid sequences of the tail and two repeats of the shaft from human Ad5 are colored in blue. The shaft sequence that was not used is not colored. The last repeat of the shaft used is colored in green. The knob of human Ad5 is also colored in gray

A series of trimeric constructs were generated as follows: the N-terminal tail and the first two shaft repeats of human Ad5 fiber fused to sTR (HA5FT), the last shaft repeat of human Ad5 fiber fused to sTR (HA5ST) and the fowl Ad1 knobless fiber fused to sTR (FA1FT) (Figure 1a). The amino acid sequences used for the constructs are indicated in Figure 1b. All fusion proteins were purified through Ni-resin by using a His-tag in the pET-28a expression vector. Owing to the reported toxicity of the polyhistidine tag in normal hepatocytes and keratinocytes^24, 25^ and lack of the optimum amount of zinc,^26^ all purified proteins were digested by thrombin, and zinc was added in the sTR samples.

**Figure 2:**
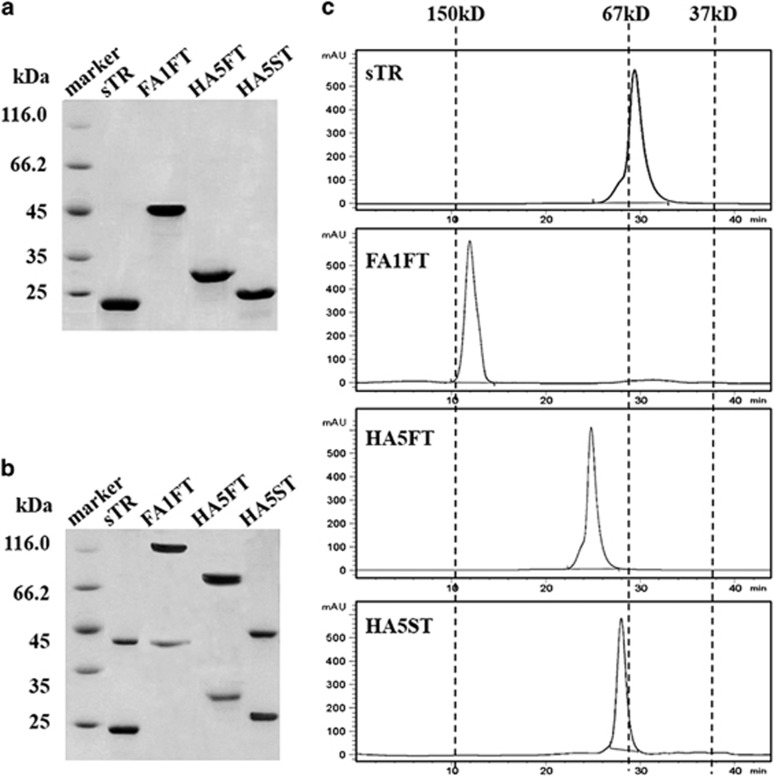
Biochemical analysis of sTR, FA1FT, HA5FT and HA5ST. Purified sTR, FA1FT, HA5FT and HA5ST were analyzed by (**a**) SDS-PAGE or (**b**) GDS-PAGE followed by Coomassie Blue staining. (**c**) sTR, FA1FT, HA5FT and HA5ST proteins were separated by HPLC TSK gel G5000, and purified IgG (150 kDa), bovine serum albumin (67 kDa) and thrombin (37 kDa) were used as standard proteins

Analysis by SDS-PAGE (Figure 2a) of the purified protein showed single protein bands with a molecular mass of ~22 kDa for sTR, ~44 kDa for FA1FT, ~30 kDa for HA5FT and ~23 kDa for HA5ST, matching the expected calculated molecular masses of 21.61, 43.48, 29.54 and 23.04 kDa, respectively. Analysis by gradually denatured SDS-PAGE (GDS-PAGE) indicated that preparations of both FA1FT and HA5FT contained more trimeric proteins than HA5ST and sTR (Figure 2b). High-performance liquid chromatography (HPLC) analysis indicated that all recombinant proteins were trimeric structures in the native buffer (pH=8.0), and the purity was above 95% (Figure 2c).

### Apoptotic activity of recombinant TRAIL fusion proteins in different cancer cell lines

**Figure 3:**
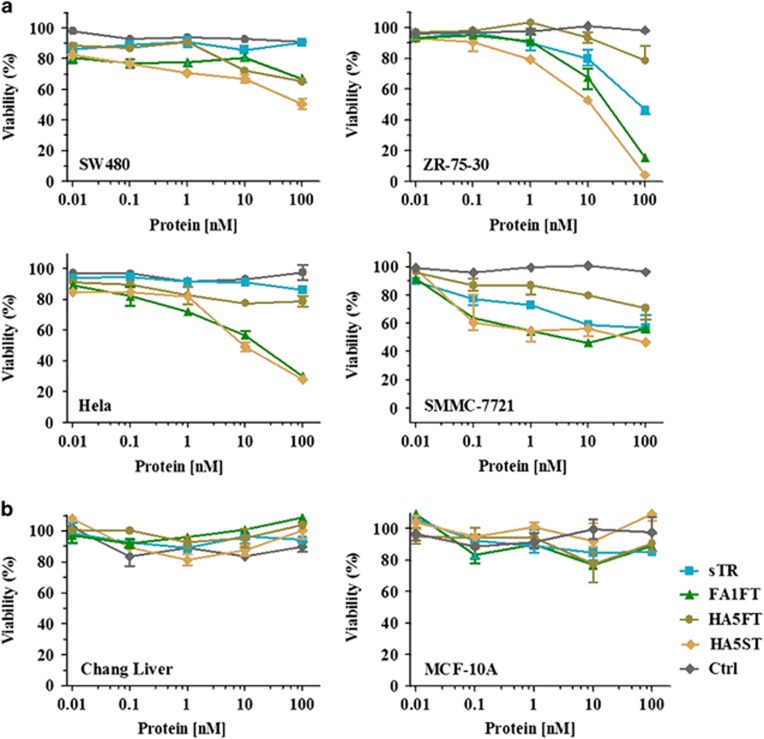
Cell viability assays. (**a**) sTR, FA1FT, HA5FT and HA5ST mediated cytotoxicity in cancer cell lines: SW480 human colon adenocarcinoma cells, ZR-75-30 human breast cancer cells, Hela human cervical cancer cells and SMMC-7721 hepatocellular carcinoma cells. All cells were incubated for 16 h with the indicated concentrations of proteins in a 96-well plate. (**b**) Cytotoxicity of sTR, FA1FT, HA5FT and HA5ST in normal human cell lines: Chang liver human hepatocytes and MCF-10A human mammary cells. All cells were incubated for 72 h with the indicated concentrations of proteins. Cell viability was determined using the MTT assay, and results were calculated as a percent relative to untreated cells. Representative killing curves from experiments with *n*=3 replicates are shown

The cytotoxicity of FA1FT, HA5FT and HA5ST fusion proteins were tested in the SW480 (human colon adenocarcinoma), ZR-75-30 (human breast cancer), Hela (human cervical cancer) and SMMC-7721 (hepatocellular carcinoma) cell lines (Figure 3a). All cells were treated with sTR, FA1FT, HA5FT or HA5ST at 10-fold dilutions from 100 to 0.01 nM for 16 h. MTT analysis showed that both 10 nM FA1FT and HA5ST induced an ∼50% level of cytotoxicity in all cells. Of note, FA1FT and HA5ST were highly sensitive to the Hela cell line compared with sTR, which has been reported to not respond to TRAIL treatment.^27^ As these cancer cells were not sensitive to HA5FT.

TRAIL has been reported to induce apoptosis specifically in various tumor cells without significant toxicity toward normal cells.^4^ We further examined whether FA1FT and HA5ST would cause cytotoxicity in normal cells; human Chang liver cells and MCF-10A human mammary cells were used for testing their effects on cell growth. As expected, FA1FT and HA5ST showed little cytotoxicity at a high protein concentration (100 nM) (Figure 3b).

**Figure 4:**
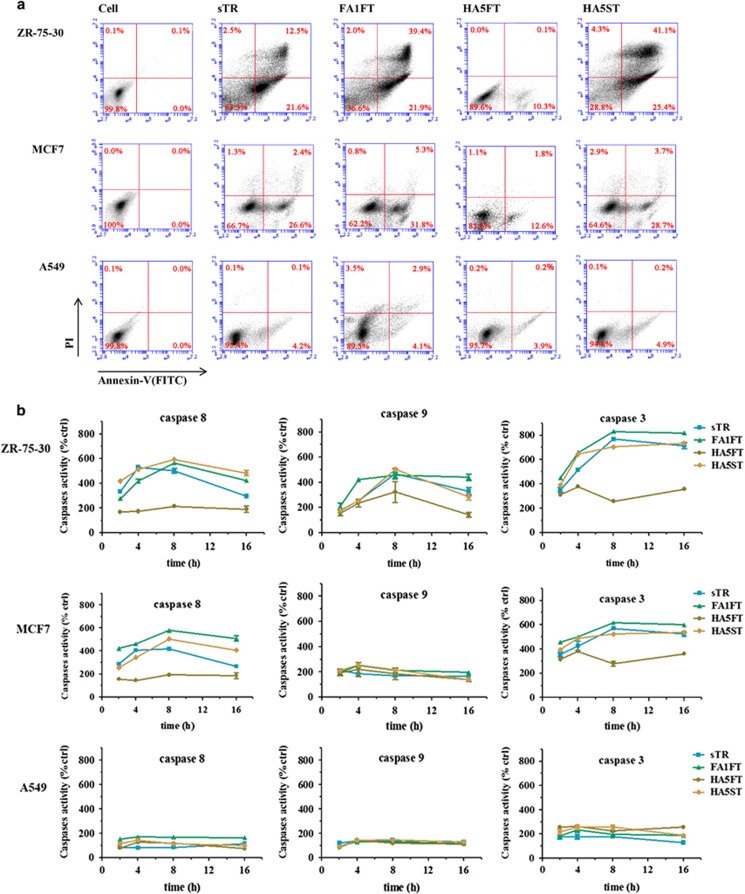
*In vitro* apoptosis activities of sTR, FA1FT, HA5FT and HA5ST. (**a**) Cells were treated with FA1FT, HA5FT and HA5ST at 10 nM for 6 h. Cells were analyzed using an apoptosis assay kit and flow cytometry. (**b**) Cells were treated with FA1FT, HA5FT and HA5ST at 10 nM for indicated times. PBS alone at the same time as control. Activities of caspase-3, -8 and -9 were measured by using substrate peptides Ac-DEVD-pNA, Ac-IETD-pNA and Ac-LEHD-pNA, respectively. The release of *p*-nitroanilide (pNA) was qualified by determining the absorbance at 405 nm, and results were calculated as a percent relative to PBS-treated cells

To further corroborate these results, FITC-conjugated Annexin V was used for detection of phosphatidylserine exposed on the membrane of apoptotic cells and for quantification of cells undergoing apoptosis. The abilities of FA1FT, HA5FT and HA5ST fusion proteins to induce apoptosis were tested in human breast cancer cell ZR-35-30 and MCF7 (TRAIL sensitive) and in human lung cancer cell A549 (TRAIL resistive).^28, 29^ Fluorescence activated cell sorting (FACS) analysis of Annexin V-stained cells showed that 61.3 and 66.8% of ZR-75-30 cells became apoptotic following treatment with FA1FT and HA5ST at 10 nM, respectively, compared with 34.1% with exposure to sTR at the same concentration (Figure 4a). In MCF7 cells, about 30–37% cells became apoptotic following a same treatment, while about 11% breast cancer cells became apoptotic with the treatment of HA5FT. In human lung cancer cell A549, the cell apoptosis was about 4.3~7% after treated with these TRAIL agents, respectively.

To confirm the apoptosis induced by these recombinant TRAIL proteins in cancer cells via caspase-dependent apoptotic pathways,^8, 9^ we tested the caspase-3, -8 and -9 activities in ZR-75-30, MCF7 and A549 cells. (Figure 4b). As expected, a continuous high level of caspase-3 and -8 activities were detected in both ZR-75-30 and MCF7 cells. No obvious signal in A549 cells could be detected. Furthermore, the outstanding apoptosis induced by TRAIL agents in ZR-75-30 cells may attribute to the high level of caspase-9 activities.

The extent of chromatin condensation was analyzed by fluorescence microscopy of cells stained with the DNA-binding fluorescent dye 4,6-diamidino-2-phenylindole (DAPI) in ZR-75-30 cultured with either FA1FT or HA5ST at concentrations from 0.1 to 100 nM (Supplementary Figure S1). All these data confirmed the high specific activity of these fusion TRAIL proteins in TRAIL-sensitive cancer cells. Since HA5FT has lower apoptosis activity, it was not tested in further experiments.

### Analysis of recombinant TRAIL fusion protein stability *in vitro*

**Figure 5:**
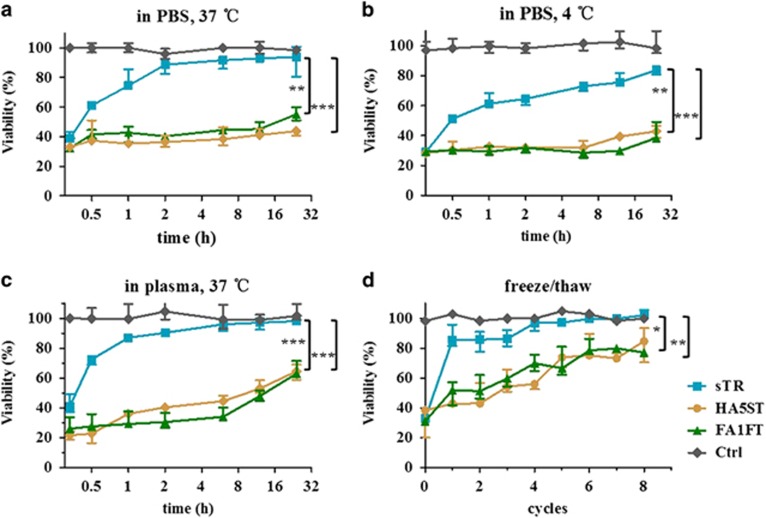
Improved characteristics of recombinant proteins *in vitro*. Killing activities of sTR, FA1FT and HA5ST were analyzed by using ZR-75-30 cells. Recombinant proteins were incubated at (**a**) 37 °C in PBS, (**b**) 4 °C in PBS or (**c**) 37 °C in plasma for the indicated times. Samples were obtained and kept on ice until used in the MTT assay (mean±S.E.M., *n*=3). (**d**) The same killing assay was performed as for samples in (**a**–**c**), but the proteins were subjected to up to 8 freeze/thaw cycles. Representative killing curves from experiments with *n*=3 replicates are shown. Statistical analysis was carried out with unpaired *t*-test, two-tailed test, *P<0.05, **P<0.01, ***P<0.001

To assess the physicochemical properties of FA1FT and HA5ST using defined *in vitro* conditions, we evaluated their stability during storage and repeated freeze/thaw cycles. All recombinant proteins were analyzed by the same killing activities with different concentrations by using ZR-75-30 cells. sTR (100 nM), FA1FT(50 nM) and HA5ST(50 nM) were incubated at 37 °C in PBS, 4 °C in PBS or 37 °C in plasma for the indicated times. When maintained in PBS, sTR readily lost a significant fraction of its initial killing potential. In contrast, FA1FT and HA5ST were unaffected by this treatment and retained nearly 100% of activity following a 24 -h incubation period (Figures 5a and b). In order to mimic the intravital condition, we analyzed the stability of FA1FT and HA5ST in plasma at 37 °C. While sTR lost its killing activity after 1 h, FA1FT and HA5ST retained 60% of the killing activity following a 24- h incubation period (Figure 5c). We also assessed the effect of repeated freeze/thaw cycles on the stability of FA1FT and HA5ST compared with sTR (Figure 5d). A rapid loss ranging between 90 and 95% of the activity of sTR was noted following only one freeze/thaw cycle compared with the non-frozen control. In contrast, FA1FT and HA5ST retained biological activity even after undergoing eight free/thaw cycles. These results indicated that the enhanced killing activity may be attributed to the improved stability of FA1FT and HA5ST, since the binding process was carried out for several hours at the intravital temperature, conditions under which sTR would have lost its bioactivity.

### Tumoricidal effects of recombinant TRAIL fusion proteins on human breast cancer in nude mice

**Figure 6:**
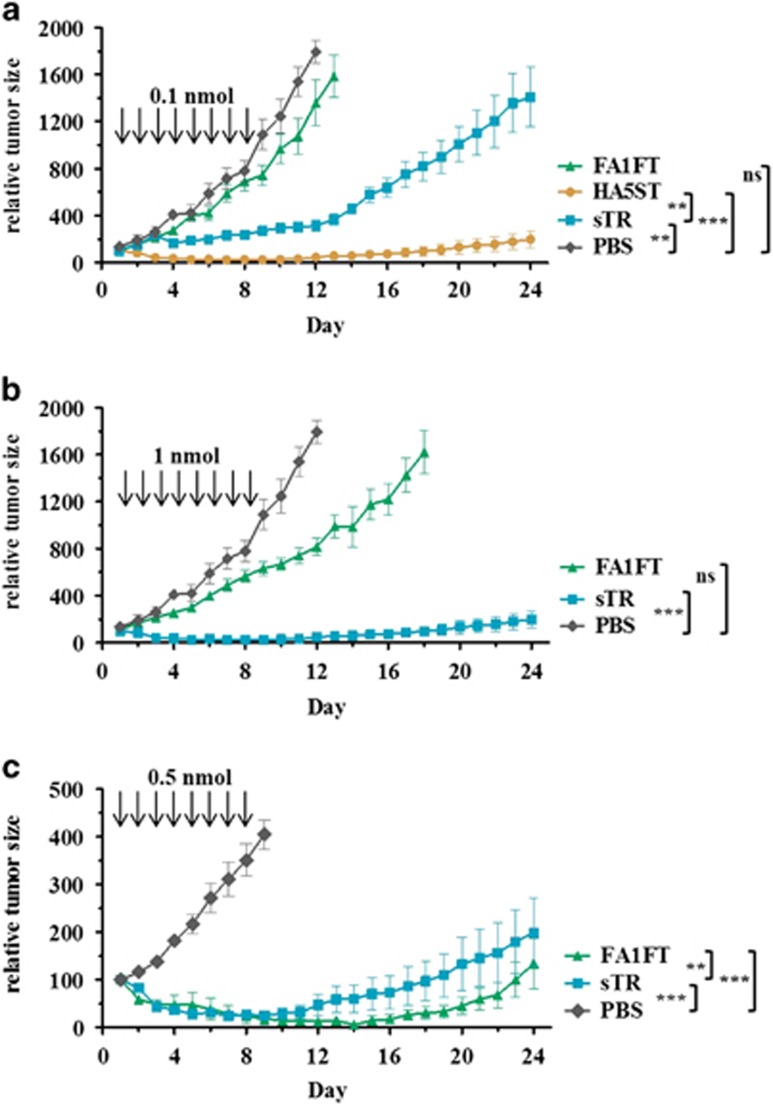
Antitumor activity in ZR-75-30 tumor-bearing mice. Nude mice were injected s.c. with 1 × 10^6^ ZR-75-30 human breast carcinoma cells. Treatment was initiated when tumors reached 100–150 mm^3^. Tumor growth suppression by sTR, FA1FT and HA5ST administered by daily injections (i.p.) from days 1 to 8, (**a**) 0.1 nmol per mouse and (**b**) 1 nmol per mouse. (**c**) Local treatment strategy. Treatment was initiated when tumors reached 200–250 mm^3^; 0.5 nmol of FA1FT and sTR were injected daily (days 1–8) in an area close to the established tumors. Statistical analysis was carried out with unpaired *t*-test, two-tailed test, **P<0.01, ***P<0.001

To investigate whether the improved tumoricidal activity of FA1FT and HA5ST observed *in vitro* would be effective *in vivo*, we investigated the antitumor activity of these proteins in nude mice carrying ZR-75-30 cells. The systemic treatment was initiated after the established solid, vascularized tumors could be clearly discerned. In the first experiment, the regimen was eight daily intraperitoneal (i.p.) injections of a low protein concentration (0.1 nmol) of sTR, FA1FT or HA5ST. Tumor growth was monitored for 24 days. With this treatment protocol, we observed significant tumor regression in the HA5ST-treated group compared not only with the sTR group but also with the control group (Figure 6a). Unexpectedly, FA1FT treatment did not induce regression of tumors. Therefore, we performed a second experiment with a high protein concentration (1 nmol) of sTR and FA1FT (Figure 6b). The treatment strategy was the same as that for the first experiment. Consequently, under the treatment conditions applied, the FA1FT group presented only a slight, but not statistically significant, tumoricidal activity, compared with the control group. A local treatment strategy was then applied by injecting 0.5 nmol of FA1FT or sTR in an area close to the established tumors. Both treatments delayed tumor growth, but FA1FT presented a stronger reduction of tumor size (Figure 6c).

### Analysis of pharmacokinetics, distribution and safety of recombinant TRAIL fusion proteins *in vivo*

**Figure 7:**
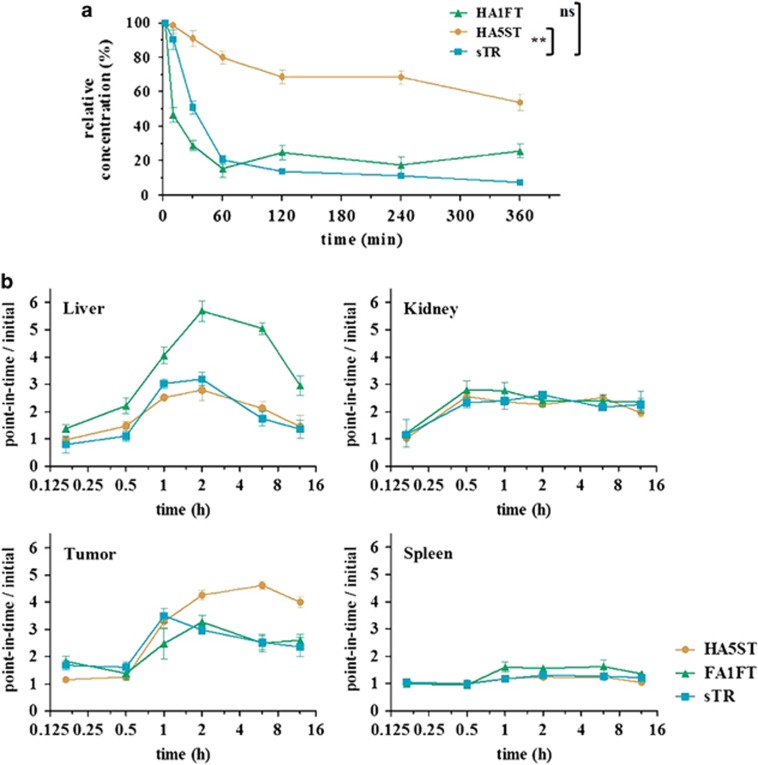
Pharmacokinetic profiles and biodistributions. (**a**) Nude mice were administered an i.v. injection of sTR, FA1FT and HA5ST (1 nmol per mouse), and blood concentrations of the recombinant proteins were monitored by ELISA. Protein concentrations were calculated as a percent relative to the 2 min samples. Each data point represents the mean value (*n*=4). Statistical analysis was carried out with unpaired *t*-test, two-tailed test, **P<0.01. (**b**) Nude mice were administered an i.p. injection of sTR, FA1FT and HA5ST (5 nmol per mouse). Supernatants of tissue homogenates were analyzed at indicated time points post injection by ELISA. Protein concentrations were calculated as a percent relative to the 10 min samples. Each data point represents the mean value (*n*=3)

To evaluate the pharmacokinetic characteristics of FA1FT and HA5ST in comparison with sTR, serum samples were collected after a single intravenous (i.v.) injection of 1 nmol into nude mice. Plasma was collected and tested for biological activity at 2 min (baseline), 10, 30, 60, 120, 240 and 360 min post injection. The relative serum concentrations plotted over time fit well to a two-phase exponential decay regression curve in both cases. Our measurements showed the presence of HA5ST in the bloodstream even 6 h after injection, suggesting that the half-life of HA5ST exceeded 1 h in mice. This figure is quite favorable when compared with a half-life of only minutes reported earlier for recombinant TRAIL.^30^ We found that both sTR and FA1FT displayed similar kinetics each with a serum half-life of ~30 min. At later time points, an obvious and significant difference in bioavailability of HA5ST was detected with a half-life of ~360 min (Figure 7a). Thus, HA5ST was more efficiently retained in the bloodstream, which may positively affect its therapeutic activity. These results demonstrate that HA5ST has more favorable pharmacokinetic characteristics compared with those of FA1FT and sTR. The improved stability of HA5ST over sTR and FA1FT, especially *in vivo*, may prove to be a significant advance for researchers as well as clinicians.

In order to investigate the reason for the insufficient tumoricidal activity of FA1FT *in vivo*, biodistributions of FA1FT and HA5ST were analyzed. At the indicated time points post injection, mice were killed, and tumors and major organs (liver, kidney, spleen and tumor) were excised. Supernatants of the tissue homogenates were analyzed by enzyme-linked immuno sorbent assay (ELISA) (Figure 7b). The results showed that tumor tissues in HA5ST-treated mice contained more of the administered fusion protein than those of FA1FT-treated mice. Moreover, the FA1FT proteins appeared to be concentrated in the liver. These results suggested that the remarkably reduced antitumor activity of FA1FT and its weak pharmacokinetic characteristics may be due to the metabolic detoxification process. The fusion proteins were detected at steady levels in the kidney, which is reasonably expected since it has been reported as a major organ for TRAIL clearance.^30^

**Figure 8:**
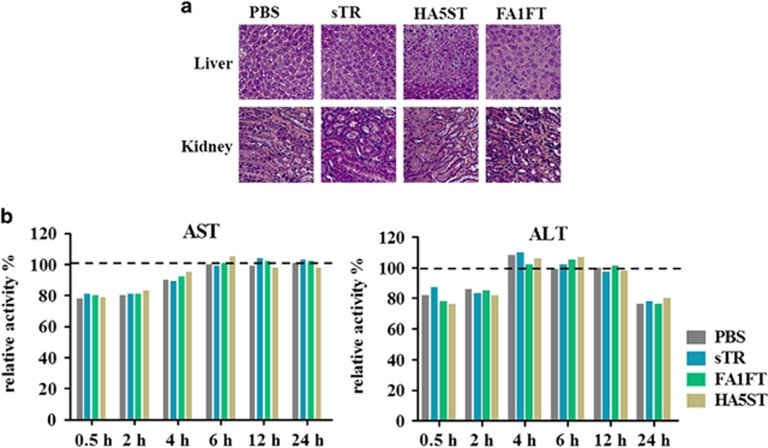
Histological investigations and hepatotoxicity. sTR, FA1FT and HA5ST were injected at 1 nmol per mouse, i.p., *n*=4. (**a**) Tissues were harvested 24 h after injection for analysis by hematoxylin and eosin staining. (**b**) Alanine aminotransferase (ALT) and aspartate aminotransferase (AST) activities in mouse serum were measured using corresponding commercial reagents. Results are expressed as a percentage relative to the level measured in untreated control mice (100% dashed line)

Histological examinations of liver tissues and kidney tissues 24 h after injecting 1 nmol of sTR, FA1FT or HA5ST showed no detectable changes by hematoxylin and eosin staining compared with the tissues of PBS-treated mice. The kidney tissues also revealed the absence of acute renal toxicity by sTR, FA1FT or HA5ST injections (Figure 8a). We further evaluated if the injections of FA1FT and HA5ST led to acute hepatotoxicity in mice by measuring levels of liver alanine aminotransferase and aspartate aminotransferase enzymes in the serum (Figure 8b). Our results indicated that the levels of both enzymes were close to the normal range. Based on these results, we concluded that our fusion proteins did not cause hepatotoxicity in mice.

## Discussion

The considerable interest in the use of TRAIL for cancer therapy is due to its unique tumor-directed apoptotic activities.^8, 9, 14, 31^ Despite its clinical advantages, however, several pharmaceutical issues, such as the instability of TRAIL under physiological conditions, its inability to form an active trimer and weak pharmacokinetic characteristics, limit its clinical applications as a cancer treatment.^30, 32, 33, 34^ To address these obstacles, researchers have focused on the development of reengineered TRAIL with improved tumor specificities and enhanced trimer-forming abilities,^35, 36^ such as leucine zipper TRAIL,^37, 38^ single-chain TRAIL (scFv-TRAIL)^39, 40, 41^ and covalently linked TRAIL trimer (TR3).^37, 42^ However, a percentage of engineered sTR proteins are produced as dimers which may be toxic to normal human hepatocytes, suggesting that more ideal genetic engineering strategies are still needed with safety as a primary consideration.

Endogenous TRAIL exists as a homotrimer, a critical requirement for its biological function. Thus, in order for recombinant TRAIL fusion proteins to function as an active homotrimer, they must mimic the natural membrane-bound form of the ligand enabling DR4- and particularly DR5-mediated apoptosis induction.^5^ In this study, recombinant human TRAIL trimers were generated based on fiber shaft trimerization elements. According to the crystal structures of the adenovirus fiber,^43^ the knob at the C-terminal of the shaft is similar to sTR in its trimeric structure. Therefore, the shaft motif would be more veritable in maintaining the stability of natural sTR compared with tagged (e.g., FLAG, His) or other types of fusion TRAIL proteins. The recombinant TRAIL variant with a fowl Ad1 fiber (FA1FT) and human Ad5 shaft (HA5ST) showed the greatest apoptotic activity in human tumor cell lines and a negligible toxic effect in normal cells (Figure 3). In addition, FA1FT and HA5ST were found to be more stable than sTR at 37 °C or after freeze/thaw cycles *in vitro* (Figure 5). Furthermore, HA5ST displayed a better pharmacokinetic profile than sTR with minimal activity loss (Figure 6). These enhanced physiological stability and better pharmacokinetic profiles than sTR resulted in improved therapeutic effects in an animal breast tumor model without detectable side effects (Figure 6). We demonstrated potent apoptosis-inducing activities of FA1FT and HA5ST, similar to that of sTR but with an enhanced stability profile *in vitro* compared with the latter. In fact, an improved half-life of HA5ST was observed, now exceeding 6 h in mice compared with a half-life of only half an hour reported earlier for recombinant TRAIL.^30^ Furthermore, HA5ST did not induce cell death in human primary hepatocytes (Figure 8). Our results clearly indicate that HA5ST has therapeutic potential for the treatment of cancers. Another important finding of our current work is the fact that the last repeat of the shaft in human Ad5 fiber could be further genetically modified as a trimerization element. We believe that this genetic approach to trimerization will extend to other TNF family members.

However, HA5FT could not induce cell apoptosis efficiently. This fusion protein was reengineered to contain N-terminal fiber tail and the shortened shaft. We speculate that HA5F domain without C-terminal repeat could not make TRAIL form an accurate trimmer, which hindered the recognition and combination between TRAIL and TRAIL receptors. Meanwhile, FA1FT was found to have substantial loss of activity *in vivo* after i.p. injection. In living animals, circulation plays an important role in the metabolism of proteins. Unlike that found with HA5ST, FA1FT did not induce tumor cell apoptosis even at high protein concentrations (Figure 6b). The local treatment results suggested that FA1FT could sufficiently induce tumor apoptosis *in vivo* (Figure 6c). The biodistribution analysis revealed that FA1FT was concentrated in the liver, which we believe was responsible for the remarkably reduced antitumor activity and the weak pharmacokinetic characteristics (Figure 7b). These unexpected results presumably was due to the shaft sequence, which is a repetitive heparin-binding motif. The human Ad5 shaft domain has been demonstrated to target to hepatic cells and cytokine release when administered through intravenous injection in mice.^44, 45^ Although most of the FA1FT proteins were detected in the liver, the fowl Ad1 shaft did not induce cell death in primary hepatocytes. On the other hand, the fiber-specific neutralizing antibodies (NAbs) are primarily against knob domain in human patients.^46, 47, 48^ In this study, all of the TRAIL compounds were engineered with knobless fiber motifs. Therefore, we speculate these recombinant soluble TRAIL proteins could not be neutralized by preexisting NAbs. Moreover, the truncate shaft motif in HA5ST with a low immunogenicity, although the regimen of eight daily continuous injections of protein may cause antibodies against shaft slightly, it will not restrain the antitumor activities of HA5ST. The therapeutic strategy of HA5FT was prefer to located injection, which may not cause an immunoreaction efficiently.

In summary, we present here a new method for generating recombinant human TRAIL based on an adenovirus shaft trimerization motif format featuring potent apoptosis-inducing activity and enhanced stability. Additional modifications demonstrated the potential of HA5ST as an investigative tool and as a platform on which to build cell-targeted anticancer therapeutics. As a consequence, the predicted advantages of such a concept would be a stronger and more sustained induction of the death receptor pathway in the targeted tumor cells. As TRAIL has been shown to augment the effects of standard therapies, a tumor-targeted HA5ST protein may further enhance the effectiveness of other chemotherapies while limiting off-target toxicities to patients. Importantly, HA5ST can be further applied in combination with small compounds in cancer therapy or for inhibiting markers of angiogenesis to gain greater effects than with either agent alone.^49, 50, 51^

## Materials and Methods

### Preparation of recombinant TRAIL proteins

Fragments corresponding to the N-terminal tail and the shaft region of Ad5 fiber were generated by PCR amplification using the fowl Ad1 fiber or human Ad5 fiber gene as a template. First-strand cDNA of TRAIL (aa 95–281) was synthesized from the total RNA of human peripheral blood leukocytes using avian myeloblastosis virus reverse transcriptase (Promega, Madison, WI, USA) and random primers.

The fusion genes were constructed by overlapping PCR. The primers were (*Nhe*I; restriction site is underlined) 5′-GCTAGCATGACCTCTGAGGAAACCATTTCTAC-3′ (sTR); 5′-GCTAGCATGGCTGACCAGAAAAGGAAGCTG-3′ (FA1FT); 5′-GCTAGCATGAAGCGCGCAAGACCGTC-3′ (HA5FT); 5′-GCTAGCATGGGTGCCATTACAGTAGGAAAC-3′ (HA5ST). The reverse primer for all constructs (*Hin*dIII; restriction site is underlined) was 5′- AAGCTTTTAGCCAACTAAAAAGGCCC-3′. All sequences were verified. Subsequently, the fragments were cloned into the pET-28a (+) expression vector (Novagen, Madison, WI, USA) by using *Nh*eI/*Hin*dIII restriction sites.

### Expression and purification of recombinant TRAIL proteins

The other constructed recombinant plasmids were transformed into competent *Escherichia coli* BL21 (DE3). The bacteria were cultured in Luria-Bertani (LB) medium (1% bactotryptone, 0.5% yeast extract, 85 mM NaCl) with vigorous shaking (220 r.p.m.) at 37 °C to a density of OD600=0.8. Thereafter, 1 mM IPTG and 50 mM ZnCl_2_ were added to induce the expression of the recombinant protein at 20 °C for 16 h. After induction, the cultures were centrifuged at 6 000 × *g* for 30 min at 4 °C. One gram (wet weight) of the 1 mM protease inhibitor PMSF was added. Supernatants were collected by centrifugation at 10 000 × *g* for 30 min at 4 °C. Harvested cells were lysed, and soluble proteins were purified by Ni-affinity chromatography (by sequential washing with 20 and 50 mmol/l imidazole and eluting with 250 mmol/l imidazole). A thrombin cleavage site was present in the N-terminal of each target protein. Thus, the His-tag was removed by digesting at 4 °C for 12 h with thrombin (10 U/mg). Finally, 0.46 mol zinc/mol protein^26^ and 1 mM DTT were added to the sTR samples.

### HPLC

The purified fusion TRAIL protein samples were applied to the HPLC TSK gel G5000 (TOSOH, Tokyo, Japan) to determine the purity and molecular mass. The column was equilibrated with two column volumes of 20 mM Na_2_HPO_4_ containing 300 mM NaCl (pH 8.0) before loading 100 *μ*l of the final purified sample at 15 ml/h.

### SDS-PAGE and GDS-PAGE

SDS-PAGE and GDS-PAGE analyses were employed according to procedures previously described^52^ using 13.5% polyacrylamide gels. Samples were denatured by boiling at 97 °C in Laemmli buffer (62.5 mM Tris-HCl, 2% SDS, 5% mercaptoethanol, 10% glycerol, 0.002% bromophenol blue) prior to SDS-PAGE analysis. GDS-PAGE was basically similar to SDS-PAGE, except that the samples were not boiled and the proteins were gradually denatured under the effect of SDS.

### Cell viability assay

The following cell lines were purchased from American Type Culture Collection (ATCC, Manassas, VA, USA): SW480, SMMC-7721, ZR-75-30, Hela, MCF7 and A549. All cell lines were cultured in Dulbecco's modified Eagle's medium or RPMI1640 (Invitrogen, Carlsbad, CA, USA) with 10% heat-inactivated fetal bovine serum (FBS; HyClone, UT, USA) plus 100 U/ml penicillin and 100 mg/ml streptomycin at 37 °C in a humidified 5% CO_2_. Experimental cells (1 × 10^4^/100 μl) were grown in a 96-well plate, incubated for 16 h and then treated with sTR, FA1FT, HA5FT and HA5ST at the indicated concentrations. Cell viability was assessed by the MTT assay according to previously described procedures.^53^

### Detection of apoptosis by flow cytometry using Annexin V staining

Early apoptotic cells were detected using the Annexin V-PI Apoptosis Kit (Beckman Coulter, San Diego, CA, USA). Cells were incubated with sTR, FA1FT, HA5FT or HA5ST for 6 h and washed with PBS. The cells were then resuspended in binding buffer, stained with Annexin V (0.6 mg/ml) and propidium iodide (PI, 5 mg/ml) for 15 min in the dark at room temperature and analyzed by two-color flow cytometry (Becton Dickinson, Franklin Lakes, NJ, USA). Data were analyzed using Cell Quest software. Annexin V-FITC fluorescence was detected in FL-1, and PI was detected in FL-3.

### Analysis of caspase-3, -8 and -9 activities

Caspase activities were measured using Caspase Activity Kit (Beyotime, C1115, CII51 and C1157, Jiangsu, China) according to the manufacturer's instructions. Briefly, cells were incubated with 10 nM sTR, FA1FT, HA5FT or HA5ST for 6 h and washed with cold PBS. The cells were then resuspended in lysis buffer and left on ice for 15 min. The lysate was centrifuged at 14 000 r.p.m. at 4 °C for 15 min. Activities of caspase-3, -8 and -9 were measured using substrate peptides Ac-DEVD-pNA, Ac-IETD-pNA and Ac-LEHD-pNA, respectively. The release of *p*-nitroanilide (pNA) was qualified by determining the absorbance at 405 nm. Results were calculated as a percent relative to untreated cells.

### DAPI analysis

Cells were incubated with sTR, FA1FT, HA5FT and HA5ST for 6 h. The cells were then fixed with 4% paraformaldehyde in PBS for 10 min at room temperature. For intracellular immunofluorescence, experiments were performed by permeabilizing the cells in 0.1% Triton X-100 for 8 min. Cells were washed three times in PBS, blocked in 10% FBS in PBS and then incubated with 1 mg/ml DAPI for 1 h at room temperature. Stained cells were washed three times in PBS before analysis. Fluorescent images were obtained using a Zeiss LSM710 confocal microscope equipped with a × 40 objective.

### ELISA analysis

For ELISA, flat-bottom Immune FEP-101 96-well plates (JET BIOFIL, Guangzhou, China) were coated overnight with 0.1 *μ*g per well of the anti-TRAIL mAb (Santa Cruz Biotechnology, Santa Cruz, CA, USA). Plates were washed three times in PBS containing 0.2% Tween20 (PBST), blocked with PBST containing 2% BSA and then incubated in triplicate with diluted plasma or diluted tissue homogenate supernatant for 2 h at 37 °C. Plates were washed again and incubated with a 1/2000 dilution of a rabbit polyclonal antibody (ab2435; Abcam, Cambridge, MA, USA) to TRAIL for 2 h at 37 °C. The plates were then incubated with a 1/5000 dilution of a horse radish peroxidase-conjugated goat anti-rabbit secondary antibody (Proteintech Group, Inc., Chicago, IL, USA). After the final wash, plates were developed with 100 *μ*l of 3,3',5,5'-tetramethylbenzidine (TMB, QIAGEN, Hilden, Germany) for 20 min at room temperature and stopped after 10 min by adding 50 *μ*l of 2M H_2_SO_4._ Analysis was performed using double wavelengths 450–630 nm with an EL × 800 Universal Microplate reader (Bio-Rad Laboratories, Hercules, CA, USA).

### Xenograft mouse models

Female BALB/c nu/nu mice (7 weeks old) were obtained from Vital River Laboratories (Beijing, China) (VRL). Human breast cancer cells (ZR-75-30; 1 × 10^6^) in log phase were implanted subcutaneously (s.c.) in the flank of each mouse. Tumor growth was monitored daily, and tumor volume was calculated by the following equation: tumor volume (mm^3^)=length × width^2^ × 0.5. After 10 days, animals with representative tumors were randomized by tumor size into four groups (*n*=10 per group, tumor volume ~100 mm^3^). Mice were given eight daily i.p. bolus doses of sTR, FA1FT, HA5ST (1 nmol/mice/day) or PBS (100 μl/mice/day). For local treatment, mice received eight daily s.c. injections of 0.5 nmol of the various TRAIL fusion proteins in PBS. Tumor growth was monitored for 20 days in the treatment groups.

### Mouse pharmacokinetics

Balb/c mice (female, 7 weeks, 20±3 g, 3 mice/group) received an i.v. injection of 1 nmol of the recombinant proteins in a total volume of 50 *μ*l. At time intervals of 5, 10, 30, 60, 120, 240 and 360 min, blood samples were taken from the tail and incubated on ice. Clotted blood was centrifuged at 3 000 × *g* for 20 min, 4 °C, and plasma samples were stored at −80 °C. Plasma concentrations of recombinant proteins were finally determined by ELISA. For data calculation, relative values of serum concentrations were analyzed with the first value (2 min) being set to 100%.

### Biodistribution assay

Balb/c mice (female, 7 weeks, 20±3 g, 3 mice/group) received an i.p. injection of 1 nmol of the recombinant proteins. At time intervals of 10, 30 min, 1, 2, 4, 6 and 12 h, the kidney, liver, tumor, lungs and spleen tissues were removed from animals and then frozen in liquid nitrogen immediately. All tissues were dissected and weighed, placed in PBS with 15 mM protease inhibitor PMSF (1 mg in 10 *μ*l) on ice. Samples were homogenized and centrifuged at 14 000  r.p.m., for 15 min, and the supernatant was aspirated for ELISA analyses.

### Statistical analysis

Statistical calculations were performed using the GraphPad Prism 5.0 statistical program.

## Abbreviations

TNF: tumor necrosis factor
TRAIL: TNF-related apoptosis-inducing ligand
Ad5: human adenovirus type 5
GDS-PAGE: gradually denatured SDS-PAGE
HPLC: High performance liquid chromatography
FACS: Fluorescence activated cell sorting
FITC: fluorescein isothiocyanate
MTT: 3-(4,5-dimethylthiazol-2-yl)-2,5-diphenyltetrazolium bromide
DAPI: DNA-binding fluorescent dye 4, 6-diamidino-2-phenylindole
i.p.: intraperitoneal
i.v.: intravenous
s.c.: subcutaneously
ELISA: enzyme-linked immuno sorbent assay
AP: alkaline phosphatase
