# Supplementary material for: Engineered adenovirus fiber shaft fusion homotrimer of soluble TRAIL with enhanced stability and antitumor activity
Source: Cell Death Dis. 2016 Jun 23;7(6):e2274–. doi: 10.1038/cddis.2016.177 (PMC5143403; doi:10.1038/cddis.2016.177)
Supplement: Supplementary Figure 1 [file cddis2016177x1.doc]

**Engineered adenovirus fiber shaft fusion homotrimer of soluble TRAIL with enhanced stability and antitumor activity**

Jingyi Yan ^1^, Lizheng Wang ^1^, Zixuan Wang ^1^, Zhen Wang ^1^, Baoming Wang ^1^, Rui Zhu ^1^, Jinpeng Bi ^1^, Jiaxin Wu ^1^, Haihong Zhang ^1^, Hui Wu ^1^, Bin Yu ^1*^, Wei Kong ^1, 2^, Xianghui Yu ^1, 2, *^


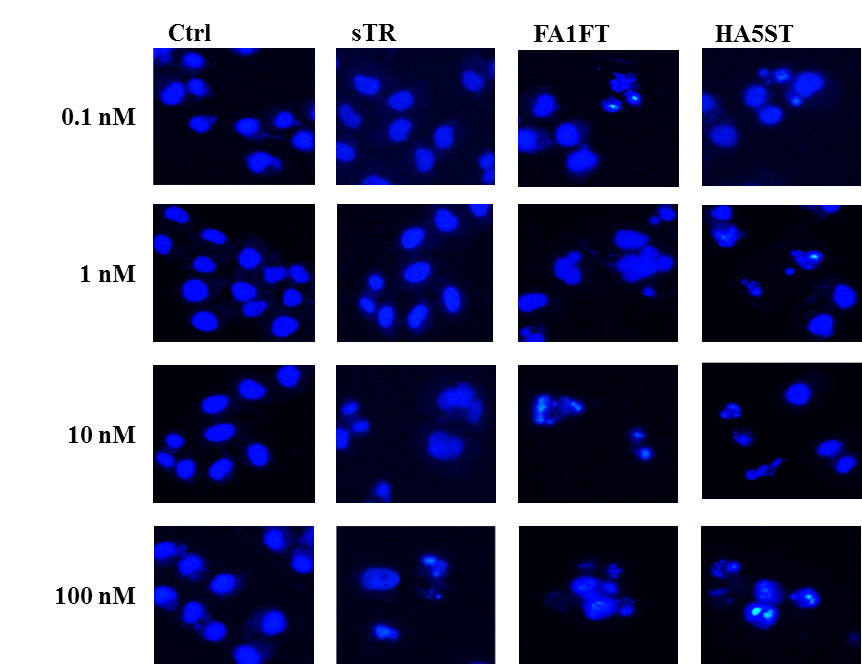


**Figure S1.** In vitro biological activities of proteins. ZR-75-30 cells were treated with FA1FT and HA5ST at 10 nM for 6 h. DAPI staining was used to determine concentration-dependent apoptotic tumor cell death.
